# Traffic particles and occurrence of acute myocardial infarction: a case–control analysis

**DOI:** 10.1136/oem.2008.045047

**Published:** 2009-06-23

**Authors:** C Tonne, J Yanosky, A Gryparis, S Melly, M Mittleman, R Goldberg, S von Klot, J Schwartz

**Affiliations:** 1 Environmental Research Group, King’s College London, London, UK; 2 Department of Environmental Health, Harvard School of Public Health, Boston, MA, USA; 3 Department of Biostatistics, Harvard School of Public Health, Boston, MA, USA; 4 Department of Epidemiology, Harvard School of Public Health, Boston, MA, USA; 5 Beth Israel Deaconess Medical Center, Boston, MA, USA; 6 University of Massachusetts Medical School, Worcester, MA, USA; 7 Institute of Epidemiology, Helmholtz Zentrum München - German Research Center for Environmental Health, Neuherberg, Germany

## Abstract

**Objectives:**

We modelled exposure to traffic particles using a latent variable approach and investigated whether long-term exposure to traffic particles is associated with an increase in the occurrence of acute myocardial infarction (AMI) using data from a population-based coronary disease registry.

**Methods:**

Cases of individually validated AMI were identified between 1995 and 2003 as part of the Worcester Heart Attack Study. Population controls were selected from Massachusetts, USA, resident lists. NO_2_ and PM_2.5_ filter absorbance were measured at 36 locations throughout the study area. The air pollution data were used to estimate exposure to traffic particles using a semiparametric latent variable regression model. Conditional logistic models were used to estimate the association between exposure to traffic particles and occurrence of AMI.

**Results:**

Modelled exposure to traffic particles was highest near the city of Worcester. Cases of AMI were more exposed to traffic and traffic particles compared to controls. An interquartile range increase in modelled traffic particles was associated with a 10% (95% CI 4% to 16%) increase in the odds of AMI. Accounting for spatial dependence at the census tract, but not block group, scale substantially attenuated this association.

**Conclusions:**

These results provide some support for an association between long-term exposure to traffic particles and risk of AMI. The results were sensitive to the scale selected for the analysis of spatial dependence, an issue that requires further investigation. The latent variable model captured variation in exposure, although on a relatively large spatial scale.

Several cohort studies provide evidence for an association between long-term exposure to ambient air pollution and cardiopulmonary mortality,^1^ ^2^ deaths from ischaemic heart disease,^3^ and acute cardiovascular events including myocardial infarction.^4^ Additional evidence is emerging that long-term exposure to air pollution or traffic indicators is associated with measures of subclinical atherosclerosis.^5^ ^6^ Furthermore, studies evaluating within-community variation in exposure have found larger effect estimates compared to between-community contrasts in exposure.^4^ ^7^ Vehicle emissions are important local sources which give rise to within-community variation in pollutants such as nitrogen dioxide (NO_2_) and primary particulate matter (PM).^8^ ^9^ Whether particles generated from traffic sources are more toxic than secondary PM is an area of active research.^10^ ^11^

**What this paper adds**

- Relatively few studies have investigated the effects of traffic emissions on cardiovascular morbidity.
- Evidence of an association is suggestive but requires additional research using improved assessment of exposure to traffic emissions.
- Results add support to a growing body of evidence of cardiovascular health effects of traffic-related air pollution.
- A latent variable approach is a useful extension of land use regression exposure models.

Previous studies investigating within-community variation in exposure and cardiovascular outcomes have used either measurements of particulate matter with aerodynamic diameter less than 2.5 μm (PM_2.5_) from monitors,^4^ ^7^ modelled concentrations from emission dispersion or land use regression models,^12^ ^13^ or indicators of residential proximity to traffic.^14^ We previously observed an association between cumulative traffic exposure near one’s residence and distance from the nearest roadway with an increased odds of acute myocardial infarction (AMI) in the central Massachusetts population using data from a population-based registry of AMI.^15^ In the present investigation, we used a latent variable approach to model residential exposure to traffic particles and assessed whether modelled long-term exposure to traffic particles is associated with an increase in the occurrence of AMI using a case–control study design.

## Methods

### Selection of cases and controls

The study population included residents of the greater Worcester (MA, USA) metropolitan area who were aged 25 years or older. Worcester is a medium-size city in central Massachusetts, with a metropolitan area population of approximately 478 000 according to the 2000 census. Cases of AMI were identified as part of the Worcester Heart Attack Study, an ongoing community-wide investigation examining changes over time in the incidence, hospital and long-term case-fatality rates of Worcester area residents hospitalised with independently confirmed AMI.^16^ Cases were admitted to any of 11 acute care general hospitals in this central Massachusetts metropolitan area during the study years 1995, 1997, 1999, 2001 and 2003. Medical records of all patients with a discharge diagnosis of AMI (ICD 9th edition code 410) were reviewed and independently validated according to pre-established diagnostic criteria described elsewhere.^16^ In brief, these criteria included a supportive clinical history, increased cardiac enzyme levels above each hospital’s normal range, and serial electrocardiographic findings indicative of AMI. At least two of these three criteria had to be met for the case to be included in the study. Information was collected from hospital medical records by trained clinicians about patients’ demographic, medical history and clinical characteristics. The present analysis is restricted to the first record of each individual case in the database; however, a case may have had a previous AMI in years other than those included in this analysis. The numbers of cases included per study year were as follows: 1995, n = 856; 1997, n = 909; 1999, n = 840; 2001, n = 1001; and 2003, n = 959.

Population controls were selected from resident lists published in 2003. Each town in Massachusetts publishes an annual list of all residents aged 17 years or older.^17^ Inclusion in the list is mandated by state law and is based on response to a mailing or visit by the town registrar. Information included in the lists varied from town to town but, at a minimum, included name, street address, sex and year of birth. Twice as many controls were selected as there were cases of AMI in the greater Worcester population. Controls were frequency matched to cases on the basis of age (in 10-year categories), sex and section of the study area such that controls were selected independently of residential location within section. The three sections were central Worcester (section 2), the northern suburbs (section 1) and the southern suburbs (section 3), and were of roughly equal population size.

Cases’ residential addresses at the time of AMI were extracted from hospital medical records and the controls’ residential addresses were collected from the resident lists. Addresses were sent to a commercial firm for geocoding (Mapping Analytics, Rochester, NY). Subjects whose residential address could not be geocoded accurately at the block group level were excluded (4%).

The study was approved by the Committee for the Protection of Human Subjects at the University of Massachusetts Medical School and the Human Subjects Committee at the Harvard School of Public Health.

### Measurement of traffic pollutants

For the purposes of estimating exposure to traffic particles in the Worcester metropolitan area, 1-week integrated concentrations of PM_2.5_ mass, PM_2.5_ filter absorbance (a proxy for elemental carbon) and NO_2_ were measured at a total of 36 monitoring sites throughout the greater Worcester area. Sampling throughout the study area was conducted from September 2003 to April 2005, although NO_2_ data were not available until December 2003. Sampling sites were identified by contacting local institutions or residents in targeted areas and asking for permission to locate a monitor on the premises. The sampling locations were chosen to provide adequate spatial coverage and to capture areas of high and low traffic intensity. All three pollutants were measured at sites where electrical power was available (n = 21). Where power was not available, only NO_2_ measurements were collected (n = 15). We used a rotating-site sampling design: during any given week, pollutant concentration data were collected at six to 10 sites for a total of 36 different locations. Samplers were placed outdoors on rooftops or at ground level, and sampler inlets were located about 2 m above the ground or rooftop surface. PM_2.5_ mass concentration was measured using Harvard Impactors (Air Diagnostics Environmental, Harrison, ME, USA) with a nominal flow rate of 4 l per minute and 2 μm pore size Pall-Gelman (Ann Arbor, MI, USA) Teflon filters. We sampled NO_2_ at each monitoring location using the Palmes tube, a passive diffusion sampler. PM_2.5_ filter reflectance measurements were taken on the PM_2.5_ filters using an EEL 43D smoke stain reflectometer (Evans Electroselenium Limited, Braintree, UK) and were converted to an absorption coefficient using the ISO 9835 formula.^18^

Additional pollution data were obtained from the US EPA’s Air Quality System for three sites within the greater Worcester area operated by the Massachusetts Department of Environmental Protection (Mass-DEP). Weekly average (n = 143) NO_2_ data were obtained from the three Mass-DEP sites between August 2003 and April 2005. In addition, 66 weekly average PM_2.5_ measurements were collected at two of the Mass-DEP sites.

### Modelling exposure to traffic particles

These air pollution data were used to estimate exposure to traffic particles using a semiparametric latent variable regression model. Latent variable modelling is similar to more widely used factor analysis. Our exposure of interest was traffic particles, which was not directly measured in the sampling campaign. Rather, exposure to traffic particles was modelled using the spatial variation common to measured NO_2_ and PM_2.5_ filter absorbance; this common variation is likely to represent sources of both pollutants such as traffic. Variation in NO_2_ that is not correlated with PM_2.5_ filter absorbance is likely due to non-traffic sources and likewise, PM_2.5_ filter absorbance, which is not correlated with NO_2_, is likely due to other sources. Because of cost constraints, we were unable to use PM_2.5_ filter absorbance monitors in all 36 locations. Less expensive NO_2_ samplers were used at all locations to provide indirect information on traffic particles. This modelling approach combined information on measured NO_2_ and PM_2.5_ filter absorbance to increase the spatial coverage of the monitoring data, rather than relying on either a small number of filter absorbance sites, or a larger network of samplers for NO_2_, which has several non-traffic sources. Exposure to traffic particles was modelled as a latent variable in the same units of PM_2.5_ filter absorbance (1E-5 m^−1^).

In addition to pollution data, the model included the following predictor variables at each monitoring location: the spatial coordinates of the sampling location and density of urbanised land according to the USGS National Land Cover Data within 1 km of the monitoring location.^19^ The spatial correlation of the latent traffic particles variable was specified using a penalised spline formulation, and the model was fit using a Bayesian Markov Chain Monte Carlo algorithm.

This approach to modelling long-term exposure assumes that the spatial distribution of emissions of traffic particles did not change during the study period. In other words, areas with high levels of traffic particles remained high over the study period. Exposure was predicted using data from the entire sampling period, such that exposure was averaged over an 18-month period of observation.

To assess the validity of the model results, we evaluated different specifications of the prior hyperparameters. The results were reasonably robust to large changes in specifications. We also evaluated model convergence by confirming the consistency of results across a wide range of starting values, conducting graphical convergence checks, as well as formal diagnostic tests.^20^ ^21^ ^22^ There was no indication of convergence problems. We checked the goodness-of-fit of the model by comparing summaries of measured PM_2.5_ filter absorbance with corresponding posterior predictive distributions. We used the approach proposed in Gelman *et al* to ensure the model adequately represented the extremes of the measured data.^23^ Additional goodness-of-fit checks indicated that posterior predictive distributions covered the measured values adequately.

### Assessment of covariates

PM_2.5_ point source emissions in 1999 were obtained from the Environmental Protection Agency (EPA) National Emissions Inventory (NEI) database.^24^ PM_2.5_ point source emissions per unit area were modelled using a kernel function to fit a smoothly tapered surface to each NEI point source using the kernel density tool in ArcGIS.^25^ The value of this function is highest at the location of the source and diminishes with increasing distance until 500 m from the source where the emissions value is set to zero. The sum of emission density from all sources at the centre of each 50 m^2^ raster cell was calculated. Subjects were assigned the estimated emission density at their residential location.

Area-based measures of socioeconomic position (SEP) were derived from the 2000 US Census at the block group level, an area smaller and, by design, more socioeconomically homogeneous than a census tract. Block groups contain 1500 residents on average, whereas census tracts contain approximately 4000 residents.^26^ We used the following census derived variables to characterise SEP: median income (median household income in 1999); percentage of persons below the federally defined poverty line; and percentage of persons aged 25 years and older whose highest degree was less than a high school diploma or its equivalent.

### Statistical analysis

We estimated the association between exposure to traffic particles and the occurrence of AMI using Proc Logistic in the SAS software package. We stratified the data according to matching factors (age, sex and section of study area) and further adjusted for spatial covariates that were considered to be potential confounders of the associations under study. We modelled traffic particles using a natural log transformation since this improved the model fit according to AIC. We included the measure of poverty in the fully adjusted model as it was the strongest predictor of AMI among the SEP variables. We evaluated potential effect modification by SEP, sex, age and section of the study area through stratified analyses.

We previously found indicators of traffic to be predictive of AMI in this study population; we therefore included an indicator of local scale traffic (the sum of the product of road segment length and estimated annual average daily traffic from the MassHighway 2002 road inventory within a 100 m radius buffer of the residence) in the same model with modelled traffic particles to explore whether the local indicator captured variation in exposure to traffic particles at a smaller spatial scale than the modelled exposure.

### Sensitivity analysis

The structure of our data involved clustering of individuals within census-defined areas. Spatial dependence may arise if individuals within census areas are more similar than they are to individuals in other areas with respect to unmeasured individual-level or contextual confounders. This lack of independence may result in incorrect effect estimates as well as standard errors. Methods for detecting and accounting for spatial dependence in the residuals of a logistic model are still in development. As a sensitivity analysis, we therefore explored the extent and spatial scale of dependence in the data using random intercept models at the block group or census tract levels. These models were fit using Proc NLMIXED.

## Results

**Figure 1:**
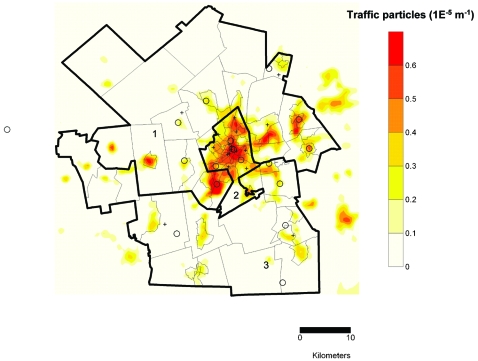
Monitoring locations and wintertime predicted levels of traffic particles (1E-5 m^−1^) within the greater Worcester area. ○ NO_2_ only monitoring locations. + NO_2_ plus PM_2.5_ filter reflectance monitoring locations. Census tract boundaries of study area are outlined in grey and the three sections of the study area are in black and numbered 1 to 3.

Measured concentrations of PM_2.5_ mass and NO_2_ as well as PM_2.5_ filter absorbance are presented according to season in table 1. Spearman correlations between site average pollution levels are also presented. Monitoring locations and modelled levels of traffic particles during winter are presented for the study area in fig 1. Levels of predicted traffic particles were highest near the city of Worcester.

**Table 1:** Air pollution measurements collected in the greater Worcester, MA, area between 2003 and 2005 and their correlations

	Pollutant		
	PM_2.5_ mass, μg/m^3^	PM_2.5_ filter absorbance, 1E-5 m^−1^	NO_2_, μg/m^3^
Number of locations	21 HSPH (256),	21 HSPH (256)	36 HSPH (523),
(measurements)	2 MA DEP (66)		3 MA DEP (143)
Mean (SD)			
Fall	8.2 (3.2)	0.55 (0.21)	11.0 (5.3)
Spring	8.5 (4.2)	0.33 (0.11)	8.9 (4.1)
Summer	11.2 (4.1)	0.45 (0.12)	7.9 (4.2)
Winter	9.2 (3.2)	0.48 (0.22)	13.4 (6.4)

		Spearman correlation (p value)	
		PM_2.5_ filter absorbance	NO_2_
PM_2.5_ mass, μg/m^3^		0.82 (<0.001)	0.74 (<0.001)
PM_2.5_ filter absorbance, 1E-5 m^−1^			0.85 (<0.001)

HSPH, Harvard School of Public Health monitoring locations; MA DEP, Massachusetts Department of Environmental Protection monitoring locations.

Demographic characteristics of cases of AMI and population controls, their area level measures of SEP, and exposures to local traffic and particles according to section of the study area are presented in table 2. Overall, cases of AMI lived in more deprived neighbourhoods and experienced higher levels of traffic and traffic particles at their residences compared to controls. Deprivation, levels of traffic and traffic particles were highest in central Worcester (section 2).

**Table 2:** Characteristics of cases of acute myocardial infarction and population controls in greater Worcester, MA, and by section of the study area

	All		Section 1		Section 2		Section 3	
	Cases	Controls	Cases	Controls	Cases	Controls	Cases	Controls
n	4565	10 277	1570	3573	2126	4907	869	1797
Demographic characteristics and AMI order								
Mean age (SD)	70 (14)	70 (14)	70 (14)	70 (14)	71 (14)	71 (14)	68 (14)	67 (14)
Male, %	57.3	56.8	60.4	60.1	54.6	54.2	58.3	57.4
Previous AMI, %	29.7	–	26.8	–	33.0	–	27.0	–
Block group socioeconomic position								
Median income, $K	48	50	60	60	38	41	52	54
Residents living below the poverty line, %	11	9	5	4	16	14	7	6
Residents with less than high school education, %	18	16	10	10	24	21	17	16
Point source PM_2.5_ emission density tons per m^2^ (SD)	0.4 (2.7)	0.4 (2.5)	0.04 (0.5)	0.04 (0.6)	0.7 (3.9)	0.7 (3.6)	0.02 (0.2)	0.04 (0.5)
Mean traffic within 100 m of residence vehicles*km	1145	1060	780	670	1635	1488	604	665
Traffic particles modelled at residence								
PM_2.5_ filter reflectance, 1E-5 m^−1^								
Mean	0.35	0.34	0.31	0.31	0.42	0.40	0.26	0.25
SD	0.13	0.13	0.13	0.14	0.10	0.10	0.11	0.11
5th Percentile	0.14	0.12	0.12	0.10	0.25	0.25	0.09	0.09
25th Percentile	0.25	0.24	0.20	0.19	0.34	0.33	0.16	0.15
50th Percentile	0.35	0.35	0.30	0.29	0.42	0.40	0.25	0.25
75th Percentile	0.45	0.44	0.41	0.41	0.49	0.48	0.34	0.33
95th Percentile	0.56	0.54	0.53	0.53	0.58	0.57	0.46	0.44

After adjusting for percentage of block group residents living below the poverty line and PM_2.5_ emitted from point sources, an interquartile range (IQR) increase in traffic particles was associated with a 10% (95% CI 4% to 16%) increase in the odds of AMI (table 3). Adjustment for other measures of SEP did not materially change the strength of the association.

**Table 3:** Relative odds of acute myocardial infarction among cases and controls (n = 14 842)

	Odds ratio* (95% CI)	
	Traffic particles	Cumulative traffic
Model		
Adjusted for matching factors	1.14 (1.09 to 1.21)	
Fully adjusted†	1.10 (1.04 to 1.16)	
Fully adjusted†+cumulative traffic	1.09 (1.03 to 1.15)	1.05 (1.02 to 1.07)
Sensitivity analysis adjusting for spatial dependence‡		
Block group		
Adjusted for matching factors	1.21 (1.08 to 1.35)	
Fully adjusted†	1.16 (1.03 to 1.30)	
Fully adjusted†+cumulative traffic	1.14 (1.01 to 1.27)	1.06 (1.03 to 1.09)
Census tract		
Adjusted for matching factors	1.07 (0.93 to 1.23)	
Fully adjusted†	1.04 (0.91 to 1.20)	
Fully adjusted†+cumulative traffic	1.01 (0.88 to 1.16)	1.06 (1.03 to 1.10)

*Per interquartile range change in natural log predicted traffic particles (1E-5 m^−1^) and natural log of cumulative traffic within 100 m buffer of residence.

†Adjusted for age, section of study area, sex, percentage block group residents living below the poverty line, and PM_2.5_ point source emissions density.

‡Block groups or census tracts included as random intercepts.

**Figure 2:**
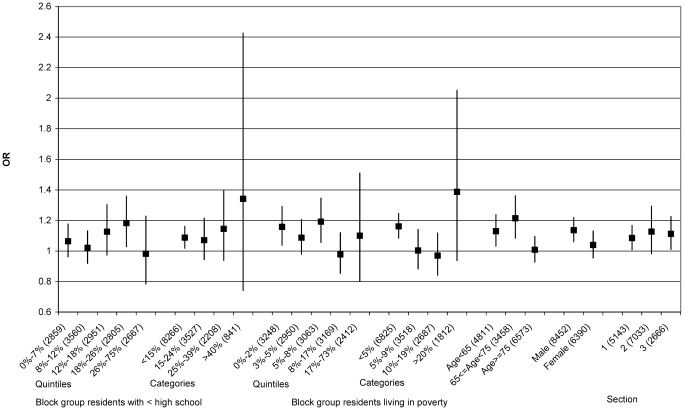
Relative odds of acute myocardial infarction and 95% CI for an interquartile range change in traffic particles according to socioeconomic position, age, sex, and study section. Traffic particles modelled as natural log. Number of subjects per subgroup included in parentheses. Odds ratios adjusted for linear age, section of study area, sex, percentage of block group residents living below the poverty line, and PM_2.5_ point source emissions density.

There was no consistent effect modification by block group level SEP when SEP was modelled as either quintiles or categories (fig 2). Traffic particles had no effect among those aged 75 years and older, although significant positive associations were observed in younger persons. The association between traffic particles and AMI was slightly greater for men compared to women.

### Sensitivity analysis

There was substantial spatial dependence in the data, and the dependence was larger at the census tract scale compared to the block group scale. The estimated variance of the random intercepts was larger in the fully adjusted model with random intercepts at the census tract level (σ^2^ = 2.2 (SE 0.49)) compared to that at the block group level (σ^2^ = 0.54 (SE 0.07)). Accounting for spatial dependence at the block group scale did not attenuate the association estimated in the conditional logistic model; however, the association was substantially attenuated in the census tract scale random intercepts model (table 3). The effect of the local traffic indicator did not change when adjusted for spatial dependence at either spatial scale.

## Discussion

We used a latent variable approach to estimate spatial variation in exposure to traffic particles within a defined metropolitan area in central New England. While this exposure assessment approach appears to effectively stratify individuals based on their risk of AMI, it captures variation in exposure on the scale of kilometres (fig 1) and likely reflects the traffic contribution to urban background rather than small scale variation in traffic particles. A recent review of the literature indicated the spatial extent of air pollution from vehicles is 100–400 m for elemental carbon and 200–500 m for NO_2_.^9^ This smaller scale variation in traffic pollutants which was not captured by the modelled exposure seems to have been captured by the indicator of local traffic. When we included the indicator of local traffic in the model with traffic particles, the effect of traffic particles remained largely unchanged: OR 1.09 (95% CI 1.03 to 1.15) per IQR increase in traffic particles, while the indicator of local traffic was also positively associated with occurrence of AMI: OR 1.05 (95% CI 1.02 to 1.07).

Although the latent variable modelling approach has several advantages, it is also subject to many of the same limitations as traditional land use regression (LUR) modelling. These include limited temporal coverage of measurements from purpose-designed sampling, reliance on passive NO_2_ sampling due to cost constraints, and incomplete traffic count data.^27^ A recent review of LUR models observed that most purpose-designed monitoring campaigns included only one to four sampling campaigns lasting between 7 and 14 days, which provide only limited information on temporal variation in exposure.^27^ Several studies, including the present study, therefore have relied on the assumption that the spatial distribution of emissions within a community remain relatively stable over time. This assumption is supported by data from a number of studies which have indicated that spatial contrasts between simultaneous measurements are relatively stable over time.^28^ ^29^ However, the lack of temporal resolution in our exposure measure limits our ability to investigate important questions regarding the timing of exposure and may lead to differential misclassification of exposure for cases in the early years of the study. We used a latent variable modelling approach to extend LUR models by pooling data on multiple measured surrogates of the unmeasured exposure of interest: traffic particles. While this approach may hold promise as a refinement to LUR exposure models, in this particular application the correlation between NO_2_ and PM_2.5_ filter absorbance was not as strong as was expected. The latent variable modelling approach is still likely to be an improvement over using only passive NO_2_ measurements; however, a denser network of PM_2.5_ filter absorbance measurements would be needed to improve the spatial variation in the predicted exposure. Exposures predicted using a similar model developed from a larger set of measurements collected in the greater Boston area had substantially more spatial variability than predictions from the model in Worcester.^30^ Distance to major road and cumulative traffic on roadways near subjects’ residence were considered as potential predictors in the exposure model. Neither measure of traffic fit the data better than degree of urbanisation. This is likely due to the inaccuracies in the traffic data, which are based on counts taken at a small number of main streets at particular times and estimated traffic flow elsewhere.

The rationale for using the latent variable modelling approach was to better identify particulate exposures from traffic sources; however, we cannot completely rule out other sources of particles that may have contributed to modelled exposure. For example, wood burning contributes to PM_2.5_ levels in the greater Worcester area in winter months as well as to NO_2_ concentrations. However, we were unable to disentangle sources other than traffic that contribute to both elemental carbon and NO_2_ with the data available from the sampling campaign. We adjusted for PM_2.5_ emission density at the residential address in order to separate the influence of traffic particles on the occurrence of AMI from particles from point sources. Misclassification in exposure to particles from point sources, resulting from the assumption of isotropic dispersion for example, could lead to mixing of the effect of particles from point sources and traffic on AMI occurrence.

Relatively few studies have evaluated the relationship between long-term exposure to air pollution and the occurrence of AMI. In the Women’s Health Initiative cohort of postmenopausal US women, a 10 μg/m^3^ increase in PM_2.5_, based on measurements from the nearest ambient monitor, was associated with a relative risk (RR) of myocardial infarction of 1.06 (95% CI 0.85 to 1.34).^4^ When the contrast in exposure was based on within-city variation, the RR was 1.52 (95% CI 0.91 to 2.51), whereas it was only 0.97 (95% CI 0.75 to 1.25) for between-city contrasts. A recent study among adult residents of Rome, Italy observed a 3% (95% CI 0% to 7%) increase in incidence of a first coronary event for every 10 μg/m^3^ increase in modelled NO_2_ exposure.^13^ In a population-based case–control study in Stockholm (AMI cases: n = 1397), there was no association between residential exposure to 30-year average NO_2_ from traffic sources and occurrence of a first AMI. The association with exposure to traffic-related PM_10_ was also not apparent.^12^ A second population-based case–control study conducted in male residents of Kaunas, Lithuania (AMI cases: n = 448) observed an OR for first AMI of 1.17 (95% CI 1.01 to 1.35) per intertertile change in annual average NO_2_ measured at monitors located in each residential district of the city.^31^ Evidence of a positive association between short-term exposure to ambient air pollution and the occurrence of AMI is more consistent.^32^ ^33^ ^34^

Emerging evidence supports the hypothesised biological mechanisms linking exposure to particles and increased risk of AMI. Controlled exposure studies in humans have demonstrated both a pulmonary and systemic inflammatory response to particulate matter.^35^ ^36^ This systemic inflammation may lead to the activation of haemostatic pathways, impaired vascular function, and acceleration of atherosclerosis, increasing the risk of plaque rupture or thrombosis and ultimately myocardial infarction.^37^ Evidence from controlled exposure studies indicates that exposure to particles increases blood levels of fibrinogen.^35^ In addition to the findings from epidemiological studies which indicate that long-term exposure to PM is associated with the extent of underlying coronary atherosclerosis,^5^ ^38^ animal studies provide supportive evidence that long-term exposure leads to acceleration of atherosclerosis.^39^

The positive association observed in the present study may also be due, in part, to bias. We lacked individual level data to adjust for SEP as well as many important risk factors for heart disease including cigarette smoking, level of physical inactivity, and unhealthy dietary habits which may be higher in more deprived neighbourhoods that also have higher levels of traffic-related air pollution. However, several risk factors for heart disease have been associated with exposure to particulate matter, and adjustment for these potential mediators of the relationship between traffic particles and AMI may not be appropriate.^40^ ^41^ Potential bias from exposure misclassification also warrants consideration. Personal exposure to traffic particles is influenced by time-activity patterns, mode of transit and workplace exposures.^42^ These aspects of personal exposure are not reflected in estimated long-term exposure outdoors at the residential address. However, it is likely that most misclassification of personal exposure to traffic particles is non-differential with respect to case status and would result in a bias towards the null hypothesis. Since we did not have data on residential history, we were unable to account for the duration of time spent at the address to which long-term exposure to traffic particles was assigned. Nonetheless, older populations such as the cases and controls in the study (mean age 70 years) move less frequently than the general population. Estimates of geographic mobility among residents of Worcester county indicate that less than 5% of residents aged 55 years and older had moved within the past year.^43^ However, the oldest individuals may have been more likely to move recently if they moved into an assisted living facility, resulting in greater misclassification of exposure among the 75 and older age strata. This may partially explain the null association observed in this age group which other studies have suggested are at elevated risk for the adverse cardiovascular effects of air pollution.^37^ A further limitation of these data is they were collected at different times, which could lead to bias due to residual confounding, exposure misclassification and control selection.

The sensitivity analysis revealed substantial spatial dependence in our data. Most methods for detecting and accounting for spatial dependence have been developed for continuous outcomes and are not easily applied to logistic or conditional logistic models. Spatial dependence in studies of environmental exposures can be complex, often resulting from a spatial mismatch between the geographic scales at which factors influencing susceptibility (eg, SEP) and environmental exposures vary.^44^ Our results were strongly influenced by the spatial scale (census tract or block group) at which we accounted for dependence. This may indicate important unmeasured compositional or contextual confounders at the census tract scale. However, modelled exposure varied on a similar spatial scale as census tracts, and it was difficult to separate the effect of exposure from census tract-scale spatial dependence. This may explain the attenuation of the association only when spatial dependence was considered at the census tract scale. Methods for assessing the degree of and controlling for spatial dependence in logistic models should be further developed, particularly in the context of air pollution epidemiology studies in which exposure is modelled from spatial predictors.

In conclusion, we observed a positive association between long-term exposure to modelled traffic particles and occurrence of AMI. The latent variable model captured variation in exposure on the scale of kilometres, whereas the indicator of local cumulative traffic appeared to capture variation in exposure on a smaller spatial scale. In study settings with relatively dense air pollution sampling, this modelling approach may provide better measures of exposure to traffic particles, and be a useful extension of LUR exposure models. The robustness of the positive association should be further investigated once methods for detecting and modelling spatial dependence in logistic models are more developed.
